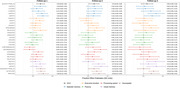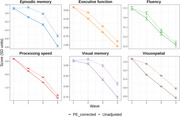# Practice effects persist over two decades of cognitive testing: Implications for longitudinal research

**DOI:** 10.1002/alz70857_106014

**Published:** 2025-12-25

**Authors:** Jeremy A. Elman, Erik Buchholz, Rouhui Chen, Mark E. Sanderson‐Cimino, Tyler R. Bell, Nathan Whitsel, Christine Fennema‐Notestine, Chandra A. Reynolds, Rosemary Toomey, Ruth E McKenzie, Michael J. Lyons, Xin M Tu, Carol E. Franz, William S. Kremen, Matthew S. Panizzon, for the Vietnam Era Twin Study of Aging (VETSA)

**Affiliations:** ^1^ University of California San Diego, La Jolla, CA, USA; ^2^ University of California, San Diego, La Jolla, CA, USA; ^3^ University of Arkansas at Little Rock, Little Rock, AR, USA; ^4^ Northwestern University, Chicago, IL, USA; ^5^ Memory and Aging Center, UCSF Weill Institute for Neurosciences, University of California, San Francisco, San Francisco, CA, USA; ^6^ Center for Behavior Genetics of Aging, University of California, San Diego, La Jolla, CA, USA; ^7^ Institute for Behavioral Genetics, University of Colorado Boulder, Boulder, CO, USA; ^8^ Boston University, Boston, MA, USA; ^9^ Merrimack College, North Andover, MA, USA

## Abstract

**Background:**

Repeated cognitive testing can boost performance due to practice effects (PEs). Follow‐up scores are rarely adjusted for PEs, but such correction can be highly important as it has been shown to result in earlier detection of MCI and increased validity based on concordance with Alzheimer's biomarkers. However, it remains unclear to what extent PEs persist across multiple follow‐ups and long durations, which are further complicated by normative age‐related decline. We examined PEs across 17 years from midlife to old age in a nonclinical sample.

**Method:**

Men (*N* = 1,608) in the longitudinal Vietnam Era Twin Study of Aging (VETSA) completed neuropsychological batteries over 4 waves: wave 1(*N* = 1,290, mean age=56); wave 2, mean ages=62); wave 3 (mean age=68); wave 4 (mean age=73). Waves 2 and 3 also included age‐matched attrition replacements. By comparing returnees’ performance to replacements being assessed for the first time, we estimated PEs for 30 tests at each wave using generalized estimating equations, accounting for normative age effects and differential patterns of missingness. We calculated unadjusted and PE‐adjusted cognitive scores (episodic memory, executive function, fluency, processing speed, visual memory, and visuospatial). At each wave we compared MCI prevalence based on unadjusted versus PE‐adjusted scores.

**Result:**

Among returnees completing all 4 assessments, we found significant PEs for 11 tests at wave 2, 8 tests at wave 3, and 5 tests at wave 4. PEs were most apparent among episodic and visual memory tests. PE‐adjusted cognitive factor scores were significantly lower than unadjusted factor scores at all follow‐ups for all domains except fluency. MCI prevalence increased up to 20% after PE adjustment (from 14% to 18% at wave 4), indicating earlier detection.

**Conclusion:**

PEs persist across multiple assessments and decades, even with long testing intervals. Importantly, even very small PEs can shift scores below the impairment threshold, resulting in earlier detection and diagnosis of MCI. Additionally, we previously showed that PE‐adjusted MCI diagnosis increases accuracy based on biomarker concordance and would dramatically reduce costs and length of clinical trials by reducing necessary sample size and increasing power. These results underscore the importance of accounting for PEs in longitudinal studies.